# Translating evidence into global impact: lessons for HIV research and policy development from the AMBITION Trial

**DOI:** 10.1016/S2214-109X(23)00412-6

**Published:** 2023-11-01

**Authors:** Joseph N Jarvis, Roger Chou, Thomas S Harrison, David S Lawrence, Charles Muthoga, Kennedy Mupeli, David B Meya, Henry C Mwandumba, Cecilia Kanyama, Graeme Meintjes, Tshepo B Leeme, Chiratidzo E Ndhlovu, Pauline Beattie, Omar Sued, Carmen Pérez Casas, Michael Makanga, Nathan Ford

**Affiliations:** 1 Department of Clinical Research, Faculty of Infectious and Tropical Diseases, London School of Hygiene and Tropical Medicine, London, UK; 2 Botswana Harvard AIDS Institute Partnership, Gaborone, Botswana; 3 Pacific Northwest Evidence-based Practice Center and Oregon Health & Science University, Portland, OR, USA; 4 Institute of Infection and Immunity, St George’s University London, London, UK; 5 MRC Centre for Medical Mycology, University of Exeter, Exeter, UK; 6 The Global Fund to Fight AIDS, Tuberculosis and Malaria, Geneva, Switzerland; 7 The Center for Youth of Hope (CEYOHO), Gaborone, Botswana; 8 Infectious Diseases Institute, College of Health Sciences, Makerere University, Kampala, Uganda; 9 Liverpool School of Tropical Medicine, Liverpool, UK; 10 Malawi-Liverpool-Wellcome Clinical Research Programme, Blantyre, Malawi; 11 Lilongwe Medical Relief Trust (UNC Project), Lilongwe, Malawi; 12 The Wellcome Centre for Infectious Diseases Research in Africa, Institute of Infectious Disease and Molecular Medicine, University of Cape Town, Cape Town, South Africa; 13 Department of Medicine, University of Cape Town, Cape Town, South Africa; 14 Internal Medicine Unit, Faculty of Medicine and Health Sciences, University of Zimbabwe, Harare, Zimbabwe; 15 European & Developing Countries Clinical Trials Partnership, Hague, Netherlands; 16 Pan American Health Organization, Washington, DC, USA; 17 Unitaid, Geneva, Switzerland; 18 Department of Global HIV, Viral Hepatitis and Sexually Transmitted Infections Programmes, WHO, Geneva, Switzerland

Translating evidence from clinical trials to routine care can take many years, particularly in low-income and middle-income countries, delaying access to life-saving or life-changing treatments. As few as one in five evidence-based health interventions are incorporated into routine use, and the average time lag between evidence availability and practice change is up to 17 years.^1^

The results of the AMBITION trial, which provided evidence supporting a simpler, safer treatment for HIV-associated cryptococcal meningitis, were published in full on March 24, 2022.^2^ A WHO rapid advice notice was released less than 1 month later, and guidelines were published in July, 2022.^3^ The rapid development of WHO guidelines facilitated incorporation of the new regimen into national guidelines in the African countries where the trial was done, and more broadly in other countries in Africa, Asia, Europe, and Latin America, with patients receiving the new treatment as part of routine care within 3 months. In this Comment, we highlight some key lessons to accelerate knowledge translation.

40 years ago, Yusuf and colleagues stated that a good clinical trial should ask an important question and answer it reliably.^4^ Cryptococcal meningitis is a leading cause of HIV-associated mortality.^5^ Until recently, the standard of care required 7–14 days of daily intravenous infusions of amphotericin B, causing significant toxicities and limiting safe use in most resource-constrained hospitals, with acute mortality rates of 40% or more.^6^

Guideline development relies on a comprehensive evidence assessment, appraised by a diverse, representative group of experts, providing an opportunity to identify crucial research gaps. The WHO cryptococcal disease guidelines from 2018 noted that simple treatments for cryptococcal meningitis suitable for low-resource settings were urgently needed.^7^

The AMBITION trial evaluated a single high dose of liposomal amphotericin B for treating for HIV-associated cryptococcal meningitis.^2^ The trial was sufficiently powered to evaluate safety and efficacy, and was done across five sub-Saharan African countries, enabling assessment of consistency of effects across different settings. The identification of the key questions was further supported by longstanding collaborations, including many African clinical researchers working in diverse health-care settings.

Guideline development at WHO follows the Grading of Recommendations, Assessment, Development, and Evaluation process, with explicit consideration of four domains: certainty of the evidence, values and preferences, balance of benefits and harms, and resource implications. Other factors such as equity and human rights, acceptability, and feasibility are also considered.^8^

Typically, trials focus only on safety and efficacy. To consider the full range of evidence-to-decision domains, guideline developers are often required to consider indirect evidence, rely on expert judgement, or await the findings of other relevant studies (such as qualitative surveys to assess acceptability), potentially adding months or years to guideline development.

The AMBITION trial took these broader considerations into account from the outset through nested cost-effectiveness,^9^ acceptability, and feasibility^10^ studies (Table). Funding to perform these analyses within the main trial was crucial to ensuring that guideline developers were provided with all the information required to formulate a recommendation.

Throughout the trial, investigators engaged stakeholders and policy makers, including ministries of health, global organisations, and the pharmaceutical industry. WHO technical staff kept in close contact with trial investigators to anticipate when trial results would be available, so that a guideline panel could be convened, with investigators sharing efficacy, costing, feasibility, and acceptability data before publication.

Engaging communities affected by HIV-associated cryptococcal meningitis enriched trial development and conduct, and was essential in showing acceptability and feasibility^10^ and helping to ensure that results were widely disseminated. Senior trial investigators contributed to national guideline-writing committees in countries where the trial was done, engagement with the Drugs for Neglected Diseases Initiative and generic drug manufactures has led to increased availability and reduced costs of the essential companion drug flucytosine, and work with Médecins Sans Frontières and other implementing partners facilitated access to and uptake of the novel treatment.

Gilead supported the preliminary phase 2 studies, donated liposomal amphotericin B for the trial, and stated their commitment to preferential pricing following trial completion, although this remains to be fully honoured. Catalytic funding from Unitaid to the Clinton Health Access Initiative established initial access to the key commodities needed for roll-out of the new treatment in African markets, a crucial factor in rapid uptake and implementation of WHO guidelines.

Extensive advocacy activities were done throughout the trial, highlighting the ongoing burden of disease and the need for new treatments,^5^ leading to an increase in global flucytosine production by generic manufacturers, substantial cost reduction, and widespread uptake in several African countries; however, considerable challenges remain. Access to both liposomal amphotericin B and flucytosine is far from universal, and the cost of liposomal amphotericin B is excessively high in several countries where exclusive distribution contracts prevent access to Gilead’s preferential pricing.^11^

The AMBITION trial shows that a single large and well done trial can rapidly and credibly translate research evidence to international clinical practice. Research teams who reliably answer important questions, simultaneously generate all evidence for decision making, and work with diverse stakeholders to advocate for change are more likely to rapidly change policy and practice and improve human health.

We would like to thank all investigators and participants in the AMBITION trial and associated substudies, community members, and the funders of the trial. The AMBITION trial was supported by a grant through the European Developing Countries Clinical Trials Partnership (EDCTP), the Swedish International Development Cooperation Agency (TRIA2015-1092), and the Wellcome Trust/ Medical Research Council (MRC) UK/UKAID Joint Global Health Trials (MR/P006922/1). Additional funding was provided by the National Institute for Health Research (NIHR) through a Global Health Research Professorship (RP-2017-08-ST2-012, to JNJ) with aid from the UK Government to support global health research. JNJ is funded by the National Institute for Health and Care Research (NIHR) through a Global Health Research Professorship (RP-2017-08-ST2-012) using UK aid from the UK Government to support global health research and received grant payments from the NIHR and EDCTP to his institution. RC received consulting fees to serve as methodologist for the updated WHO guideline on cryptococcal meningitis. TSH and DSL received support for the underlying AMBITION trial to institution from EDCTP, MRC, and Wellcome Trust, UK aid from the UK Government to support global health research, and provision of drug for the underlying AMBITION trial from Gilead. All other authors declare no competing interests. The views expressed in this publication are those of the authors and not necessarily those of the NIHR or the UK Department of Health and Social Care. The funders had no role in study design, data collection and analysis, decision to publish, or preparation of the manuscript. The writing of this Comment was supported by a grant from the Bill & Melinda Gates Foundation. We are grateful to Susan L Norris and Rebekah Thomas for providing comments on an earlier draft.

## Figures and Tables

**Table:** Evidence to Decision Table for the treatment of cryptococcal meningitis

Criterion*	Source of information	Judgement
Priority of the problem	Published estimate of global disease burden^5^	High priority: 152 000 cases of cryptococcal meningitis, resulting in 112 000 cryptococcal-related deaths
Balance of benefits and harms	Phase 3 clinical endpoint trial (the AMBITION trial)^2^	Benefits outweigh harms: non-inferior mortality; fewer adverse events
Acceptability to key stakeholders	Ethnographic study (the LEOPARD study)^10^ embedded within the phase 3 clinical trial. In-depth interviews with trial participants and providers, direct observations	Highly acceptable from the perspective of participants and providers
Feasibility of implementation	Ethnographic study (the LEOPARD study) ^10^ embedded within the phase 3 clinical trial. Direct observations and in-depth interviews with trial investigators	Feasible: Simpler to administer resulting in fewer side effects and preferred to the previous standard of care
Resource requirements	Economic evaluation^9^ embedded within the phase 3 clinical trial	Cost-effective at a low incremental cost-effectiveness ratio; potentially cost saving in real-world implementation
Impact on health equity	Equity considerations provided during the guideline meeting	Improves equity

*For a guideline panel to make a recommendation for or against a given intervention, each of these criteria listed in the first column needs to be considered. Gathering information early to inform these recommendations is crucial to accelerating guideline development*.
